# Nutritional and Functional Values of Grape Seed Flour and Extract for Production of Antioxidative Dietary Supplements and Functional Foods

**DOI:** 10.3390/molecules30092029

**Published:** 2025-05-02

**Authors:** Yavor Ivanov, Milka Atanasova, Tzonka Godjevargova

**Affiliations:** Department Biotechnology, University “Prof. Dr. Asen Zlatarov”, 8010 Burgas, Bulgaria; qvor_burgas@abv.bg (Y.I.); milka_88@abv.bg (M.A.)

**Keywords:** grape seed flour, grape seed extract, polyphenol content, nutritional value, inhibition, α-amylase, α-glucosidase, pancreatic lipase, in vitro digestion

## Abstract

The potential of the seed flours and extracts of the red grapes Pinot Noir and Marselan for application as food additives and antioxidative dietary supplements was determined. The differences between the quality characteristics of the seeds, flour, and extracts of the two grape varieties were examined. The polyphenol composition and antioxidant potential of the two extracts were compared. The extracts were rich in polyphenols, especially flavonoids (52.01 mg QE/g DW) and procyanidins (152.18 mg CE/g DW). The nutritional composition of the flours and extracts was determined. It was found that the content of ash, crude protein, crude fat, and total dietary fibers in the flour from the studied grape seeds was about 1.5 times higher than that in the extracts. Only the carbohydrate content was 2–2.5 times higher in both extracts compared to that in the flours. The content of macro and microelements in the seed extracts and in the flours was high. The inhibitory potential of the two seed extracts on three key enzymes affecting diabetes and obesity—α-amylase, α-glucosidase, and pancreatic lipase—was studied. The highest degree of extract inhibition against α-glucosidase was determined (IC_50_—2.53 ± 0.24 µg/mL). A real inhibitory assessment of the extracts was made by implementing an in vitro digestion simulation method. It was found that the percentage of inhibition of the enzymes with the digested extract was higher compared to those with the undigested extract in buffer and salt solution. Our study proves that the high content of flavonoids and procyanidins in the two extracts determines their high inhibitory capacity against the three enzymes and their potential for managing diabetes and obesity.

## 1. Introduction

Plants have been utilized as a natural source of remedies in healthcare for millennia [1]. They have long been used as folk herbal medicines to treat various disorders, and their different bioactive compounds have inspired the development of new drugs [2]. The development of new methods for extracting biologically active substances and examining the individual compounds contained in plants has led to the development and application of new plant-based medical products such as supplements, drugs, and therapeutic products. One of the oldest and most cultivated plant species is the grapevine (*Vitis vinifera* L.) [3]. Grape is among the most preferred plant sources due to its high content of biologically active substances—polyphenols, polysaccharides, proteins, vitamins, metals, etc. [4]. These substances not only have nutritional value but also prominent biological activity. Polyphenols are one of the most important components due to their valuable biological properties. Their content varies in different parts of the grape. The highest content of polyphenols is found in grape seeds 60–70%, while in the pulp and skin, the content is less than 10% and 30–40%, respectively [5]. In wine production, grape pomace, which mainly contains grape seeds and skin and is a potential environmental pollutant, is discarded as a by-product. Recent trends show that due to the high content of polyphenols in grape seed extracts (GSEs) and flours, as well as from an ecological and economic point of view, there is considerable interest in it [6,7,8]. The main phenolic compounds that predominate in grape seeds include catechin; epicatechin; gallic acid; and procyanidin B1, B2, and B3. [9,10,11]. These phenolic compounds have a high antioxidant and antimicrobial effects. Due to these properties, they have a significant positive effect on human health and exhibit antiatherosclerotic, anticancer, antidiabetic, and anti-inflammatory properties, both in humans and in animal models [12,13,14].

Polysaccharides, polyunsaturated fats, proteins, metals, and vitamins present in grape seeds are also intriguing bioactive substances [15,16,17]. That is why in recent years the market for grape seed-based supplements and food additives has been constantly growing [18]. There are a significant number of publications that have presented the nutritional composition of GSE and flour [17,19,20]. The values of the studied nutritional parameters (ash content, protein, fat, moisture, fiber, and carbohydrates) presented by different authors vary due to differences in grape species, soil, climate, and geographical location [4]. Usually, seed flour is used as an ingredient for the preparation of functional foods [18,21]. Grape seed extracts are also used as food additives and as supplements [22,23]. Due to the extraction process, the polyphenol content and antioxidant capacity of grape seed extracts are much higher than those in the flour, and they find an extremely wide application as supplements [24]. Grape seed extracts are generally recognized as safe (GRAS) and are officially approved by the Food and Drug Administration (FDA) [25].

It is a very important task to prove the biological activity of grape supplements. Various in vivo studies have shown that grape seed extracts containing dietary phenolic compounds are good inhibitors of α-amylase, α-glucosidase, and pancreatic lipase [9,26]. It is known that one of the main ways to control diabetes is by inhibiting salivary α-amylase, which hydrolyzes starch to oligosaccharides and disaccharides. These intermediates are further broken down to monosaccharides (glucose and fructose) by the enzyme α-glucosidase and absorbed into the blood stream [27,28]. Another enzyme that is of great importance to obesity is pancreatic lipase, which breaks down lipids—mainly dietary triacylglycerides—to monoglycerides and free fatty acids, which can be easily absorbed into the circulatory system [29,30]. The inhibition of pancreatic lipase reduces fat absorption in the small intestine and prevents excessive weight gain [31]. The degree of enzyme inhibition activity depends on the type of grape, as grape seed extracts have different polyphenol contents [32]. It is believed that grape seed’s inhibition of these three enzymes can lead to good management of diabetes and obesity [33,34].

To obtain a realistic inhibitory assessment, it is necessary to conduct in vitro studies and to monitor the influence of the digestive tract on the biological properties of grape seed extracts. Several in vitro models simulating the digestive environment have been described [35,36]. By including gastric and key intestinal enzymes, and by adjusting factors such as pH, temperature, and salt composition, key phases of the digestive process are mimicked. It has been shown that such methods can be effective for assessing the bioavailability of a wide range of drugs, nutrients, and phytoconstituents [36]. Several publications indicate that grape seed extracts retain their phenolic content and antioxidant capacity under gastrointestinal conditions [37,38], but there are no publications comparing the degree of inhibition of the enzymes α-amylase, α-glucosidase, and pancreatic lipase in buffer, in salt solutions (mimicking the gastrointestinal tract), or under the conditions of a gastrointestinal model.

In our previous paper, the high polyphenol content and antioxidant and antimicrobial potency of the seed extracts of Pinot Noir and Marselan red grape varieties were presented [39]. The nutritional values and macro- and microelements of Pinot Noir and Marselan seed flours and extracts were not studied. It was necessary to investigate these parameters for a complete evaluation of the application possibilities of these two extracts as food additives and supplements.

Through this research, we wanted to highlight and provide the distinctive qualities (physicochemical characteristics, polyphenolic contents, antioxidant potentials, nutritional values, macro- and microelement contents, and functional values) of grape flours and GSEs obtained from two varieties of red vines, Pinot Noir and Marselan. The biological activity of these extracts was proved by investigating their inhibitory effect on α-amylase, α-glucosidase, and pancreatic lipase in the conditions of a model simulating the gastrointestinal tract. To our knowledge, there is no research data for the inhibition activity of Pinot Noir and Marselan GSEs against the studied enzymes. Our results offer new perspectives and research opportunities for the incorporation of these extracts into different functional foods, or for their use as supplements to manage diabetes and obesity.

## 2. Results

### 2.1. Polyphenol Contents of Pinot Noir and Marselan Grape Seed Extracts

The studied Pinot Noir and Marselan grapes are grown in the Danube region near the city of Silistra, Bulgaria. The grape seeds used in this study were from pomace from the vinification of red wines from *Vitis vinifera* L. cv. Pinot Noir and Marselan grapes (Pink Pelikan Winery Ltd., Silistra, Bulgaria).

**Figure 1:**
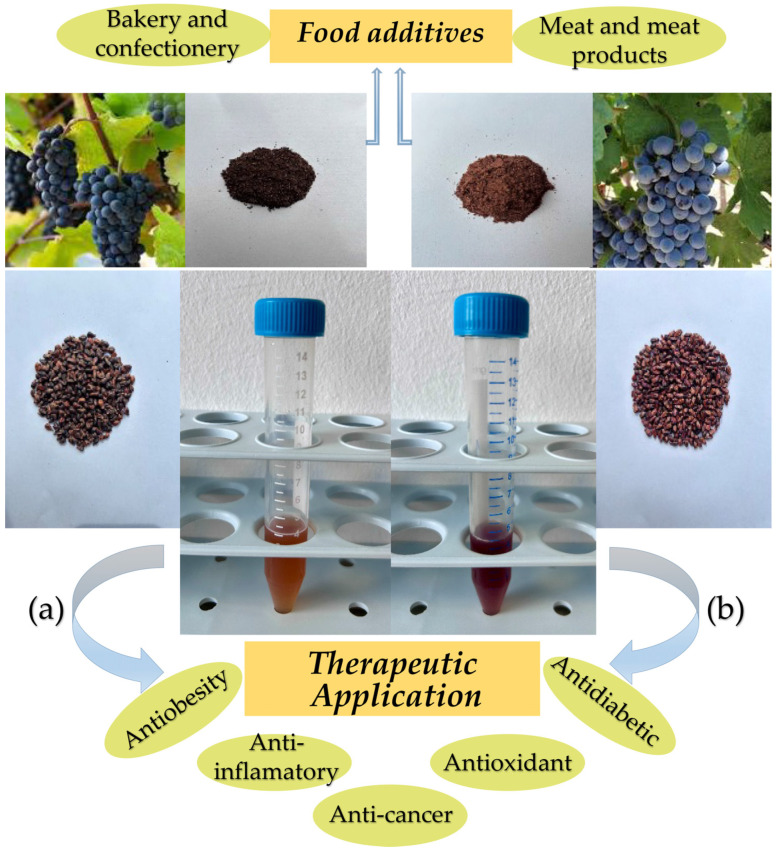
Pinot Noir (**a**) and Marselan (**b**) grapes, seeds, flours, and extracts, and their possible applications.

Figure 1 presents the grapes, seeds, flours, and extracts of Pinot Noir and Marselan and their application possibilities.

Pinot Noir belongs to the Eurasian species and exhibits early ripening, thin skin, and a purple-black color. Marselan is late-ripening, with large ears and blue-black skin. From Figure 1 it can be seen that the color of Marselan seeds is slight redder than that of Pinot Noir seeds. The grape seeds can be used as minced flour and as extract. The grape powder has a particle diameter of 2.5–22.5 µm. The color trend for the two grape seed powders is the same as for the seeds. The extraction of grape seed flour was carried out with 70% ethanol solution, by stirring at room temperature. In our previous paper, the influence of the type of solvent (70% aqueous solution of ethanol, methanol, or acetone) on the extract yield was studied [40]. The highest yield (15%) and highest total phenol content were obtained with 70% ethanol (112 mg GAE/g DW). Usually, for use of the extract in the food industry and in medicine, it is preferable to use ethanol as the extracting agent because it is safer for health. The influence of ethanol solution concentrations (50, 70, and 90%) was also studied. It was found that the 70% ethanol concentration was optimal. The color of the obtained Marselan seed extract is slight redder than that of Pinot Noir (Figure 1).

The colors of the flours and extracts of these two types of grapes were measured using a Konica Minolta device, CR-410 Chroma Meter, Konica Minolta, Osaka, Japan. The color parameters light (L*), redness (a*), and yellowness (b*) are presented in Table 1.

The redness of Marselan flour is 1.55 times higher than Pinot Noir flour redness, but for the extract, it is 3.3 times higher. The color of Pinot Noir flour and extract has less redness and would be more suitable for use as a food additive. Usually, seed flours are used as an ingredient for the preparation of functional foods, but grape seed extracts are used as supplements and as food additives (Figure 1).

**Figure 2:**
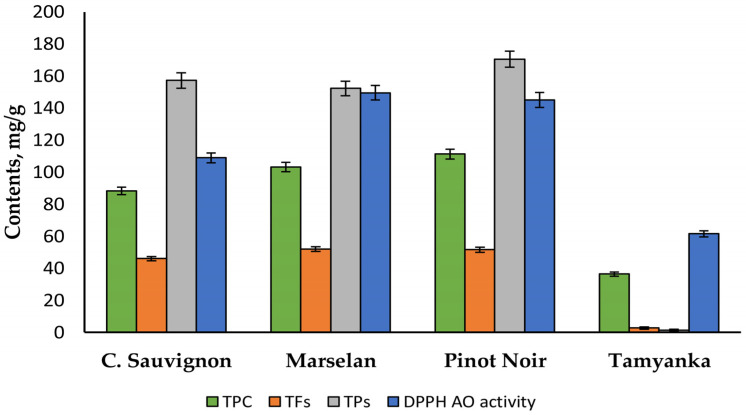
Total phenol content (TPC, mg GAE/g DW), total flavonoids (TFs, mg QE/g DW), total procyanidins (TPs, mg CE/g DW), and DPPH antioxidant activity (mg TE/g DW).

The polyphenol contents and individual compositions of the seed extracts from three red grape varieties (Pinot Noir, Marselan, and Cabernet Sauvignon) and one white grape (Tamyanka) were investigated in our previous paper [39]. The polyphenol contents and antioxidant capacities of these seed extracts are presented and compared in Figure 2.

TPC, TFs, TPs, and DPPH antioxidant activity was determined by spectrophotometrically methodologies, as presented in paper [39]. The Pinot Noir and Marselan grape seed extracts have higher total phenol content (TPC), total flavonoids (TFs), total procyanidins (TPs), and DPPH antioxidant activity than the other two types of grape extracts. The results for the Pinot Noir grape seed extract were as follows: TPC—111.22 mg GAE/g (gallic acid equivalents), TFs—51.5 mg QE/g (quercetin equivalents), and TPs—170.45 mg CE/g (catechin equivalents). The data for the Marselan grape seed extract were similar: TPC—103.24 mg GAE/g, TFs—52.01 mg QE/g, and TPs—152.18 mg CE/g. The DPPH antioxidant activity (mg Trolox equivalents) of the Pino Noir and Marselan extracts was 1.5 times higher than that of the Cabernet Sauvignon seed extract and 2.5 times higher than that of the Tamyanka seed extract. The individual compounds of Pinot Noir and Marselan seed extract were also studied by HPLC in our previous paper [39]. The following bioactive compounds were detected in these extracts: gallic acid; catechin; epicatechin; epigallocatechin gallate; procyanidin B_1_, B_2_, and B_3_; procyanidin C_1_; oenin; and quercetin. Comparison with results from other authors showed that these two types of seed extracts have a high polyphenol content [9,10,41]. For this reason, the Pinot Noir and Marselan extracts were selected for this investigation.

The polyphenol content, antioxidant activity, and antimicrobial capacity of Pinot Noir and Marselan seed extracts are very well studied, but their approximate composition has not been investigated. For a more complete study of the application possibilities of grape seed flours and extracts, it was necessary to study not only polyphenol contents, but also nutritional values and macro- and microelement contents.

### 2.2. Determination of Nutritional Value of Pinot Noir and Marselan Grape Seed Flours and Extracts

In order to make an accurate assessment of the nutritional value of the seed flours (GSFs) and extracts (GSEs) of Pinot Noir and Marselan grape varieties, their ash, moisture, protein, fat, fiber, and carbohydrate content was investigated. It was found that the chemical compositions of the flours and seed extracts of the two grape varieties are different (see Table 2).

The content of ash, protein, fat, and fiber in GSF is higher than in GSE. Only total carbohydrates are higher in GSE than in GSF. All the results between the two types of flours and extracts are similar.

### 2.3. Determination of Mineral Profile of Pinot Noir and Marselan Grape Seed Flours and Extracts

The mineral contents of the studied grape seed flours and extracts were determined using Thermo Scientific (Waltham, MA, USA) iCAP Q ICP-MS. The studied elements have a strong influence on human health and play an important role in the formation of bones and teeth, enzyme structure, muscle contraction, kidney function, proper heart rhythm, nerve signaling, etc. [42]. The mineral compositions of grape seed flours and extracts are summarized in Table 3.

In grape flour samples, the highest content is potassium (5108.01 ± 122.64 mg/kg), followed by phosphorus (3821.53 ± 263.95 mg/kg), calcium (1853.80 ± 74.50 mg/kg), and magnesium (1464.12 ± 92.96 mg/kg). For the extracts, this order is slightly altered: in first place is potassium (5279.99 ± 113.16 mg/kg), followed by phosphorus (3693.10 ± 25.08 mg/kg) and, in third place, with slightly different values, sodium (542.04 ± 22.32 mg/kg) and magnesium (461.87 ± 15.95 mg/kg). In general, most of the metals have a lower content in the extracts than in the flours, probably due to their weaker extraction during the processing of ground grape seeds with 70% ethanol. An exception to this trend is observed in potassium and sodium in both types of grape seed extracts, and also in phosphorus, but only for the Pinot Noir grape seed extract. A significant percentage of the population is deficient in the microelements valuable for health, like zinc, copper, and iron. It was determined that the zinc content in Pinot Noir flour is 19.75 ± 0.56 mg/kg and 12.09 ± 0.55 mg/kg in Marselan flour. Its content in the corresponding extracts is slightly lower—14.72 ± 0.25 mg/kg and 3.19 ± 0.22 mg/kg, respectively. Copper is also a very important trace element, necessary for the catalytic action of some enzymes. Its content in flour and extract is similar to zinc. In contrast, the iron content is higher. Pinot Noir flour contains 160.89 ± 2.91 mg/kg of iron, and Pinot Noir extract 119.07 ± 2.08 mg/kg of iron.

### 2.4. Determination of Inhibitory Effect of Pinot Noir and Marselan Grape Seed Extracts on Salivary α-Amylase, α-Glucosidase, and Pancreatic Lipase

**Figure 3:**
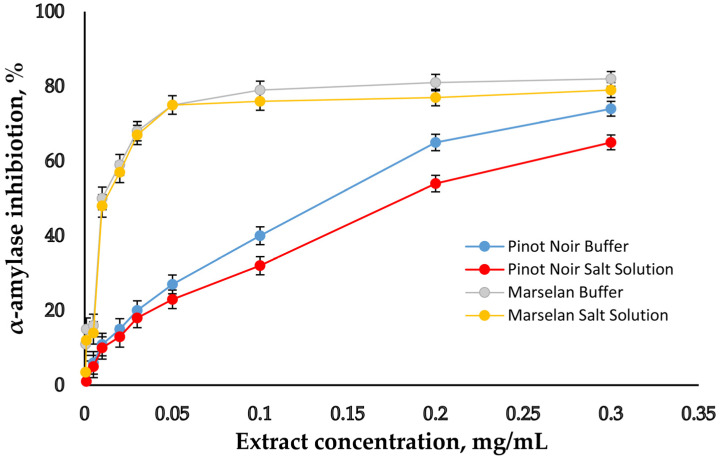
α-amylase inhibitory activity of GSE. Bar indicates Mean ± SD (*n* = 3).

**Figure 4:**
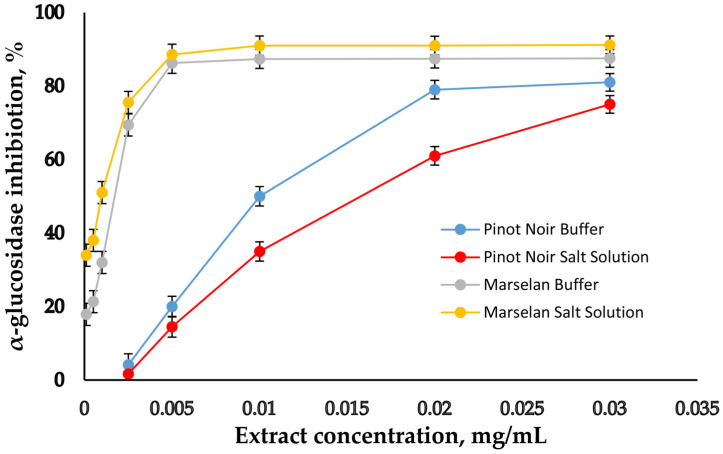
α-glucosidase inhibitory activity of GSE. Bar indicates Mean ± SD (*n* = 3).

**Figure 5:**
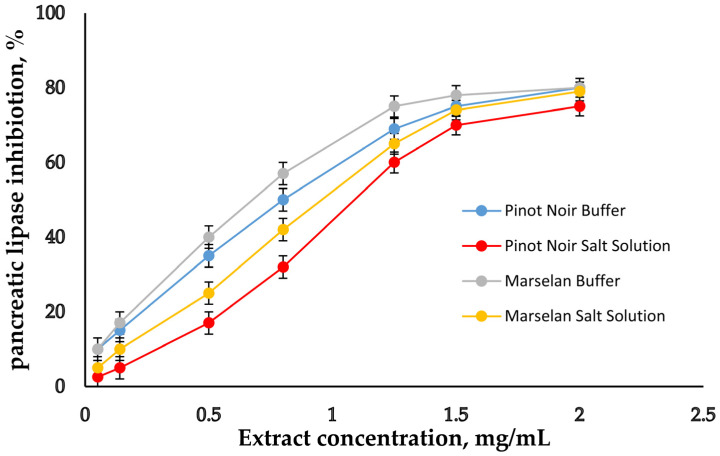
Pancreatic lipase inhibitory activity of GSE. Bar indicates Mean ± SD (*n* = 3).

In order to make a convincing assessment of Pinot Noir and Marselan grape seed extracts as supplements, experiments on the inhibitory potential of GSE on salivary α-amylase, α-glucosidase, and pancreatic lipase were conducted. First, the experiments were carried out in buffer solutions to provide the optimal conditions for determining the activity of each enzyme (optimal pH and optimal temperature). The activities of the enzymes were selected depending on their typical activity in the native states of the corresponding digestive phases [9,25,26]. The concentrations of the two extracts were varied, and their inhibition potential against the studied enzymes was determined. On Figure 3, Figure 4 and Figure 5, the inhibitory curves of GSE are presented.

The concentrations of the extracts at which 50% inhibition (IC_50_) of the individual enzymes occurred were calculated on the base of inhibition curves and are presented in Table 4.

The lowest IC_50_ value was obtained for α-glucosidase, followed by α-amylase and finally for pancreatic lipase. For comparison, the same experiments were performed in salt solution, with a composition similar to the in vitro model for simulating the digestive environment, without digestive enzymes in it [34,37]. The aim of this study was to monitor the influence of salts on the enzymes’ inhibition. In the experiments with salt solution, the enzymes were dissolved in buffer with an optimal pH for the respective enzyme, and then added to the salt solution with a pH of 7. The results show that both in buffer and in salt solution, α-glucosidase is much more sensitive to GSE’s inhibition activity than the other two enzymes. When comparing the results obtained in buffer and in salt solution, a weaker degree of inhibition of all three enzymes in salt solution is noted, as are correspondingly higher IC_50_ values. Obviously, salts do not have a positive effect on enzymes’ degree of inhibition. The IC_50_ results were compared with those of commercial synthetic drugs used for diabetes (Acarbose) and obesity (Orlistat). In Table 4, it can be seen that the inhibitory potential of these two drugs is weaker compared to the two extracts.

### 2.5. In Vitro Digestion

In vitro experiments were conducted to study the impact of digestive conditions on the biological properties of the studied grape extracts, and, specifically, on their inhibitory potential against the three enzymes. By including key oral, gastric, and intestinal enzymes and by regulating factors such as pH, temperature, and salt concentration, the basic phases of the digestive process were successfully imitated. The experiments took into account the localization of digestive enzymes in the individual phases. The oral phase included salivary α-amylase, the gastro phase included pepsin, and the intestinal phase included pancreatin. The influence of digestive enzymes and salts on the inhibitory potential of GSE that was sequentially passed through the three digestive phases toward the studied enzymes was monitored. The degree of inhibition of the three enzymes was determined by separating an aliquot part of the solution after the incubation of the extract in each digestive phase. The activity of the three enzymes was determined using the same methods as for determining the activity of the enzymes in buffer and in salt solution. The aim was to assess the actual, real antidiabetic and anti-obesity potential of GSE under physiological conditions. The results obtained for the degree of inhibition of salivary α-amylase, α-glucosidase, and pancreatic lipase from digested GSE are presented in Table 5 and were compared with the results obtained with undigested GSE in salt solution.

In an aliquot of the solution from the first oral phase, α-amylase inhibition was determined. Its degree of inhibition was similar to the previous result in Table 4, given that in the oral phase, only α-amylase was incubated with GSE and salt solution for 10 min. For this reason, no result for α-amylase is presented in Table 5. After the incubation of the gastro and intestinal solutions, the aliquots were separated to determine the degree of inhibition of α-glucosidase and pancreatic lipase. In these cases, the added enzymes—pepsin in the gastro phase and pancreatin in the intestinal phase—affected the extract during incubation. A comparison was made of the inhibitory potential of the digested extracts and the undigested extracts dissolved in salt solution. Table 5 shows that the percentage inhibition of α-glucosidase with undigested Pinot Noir extract dissolved in salt solution was 1.5 times lower compared to the digested extract. In the case of the Marselan extract, this difference was smaller, but again, in the digested extract, the percentage of enzyme inhibition was higher. The higher results with digested Marselan extract compared to digested Pinot Noir extract are analogous to the results in buffer and salt solution. This trend was repeated for pancreatic lipase.

## 3. Discussion

The polyphenol content of Pinot Noir and Marselan seed extracts were determined. The results in Figure 2 show that the total phenol content (TPC), the total flavonoids (TFs), and the procyanidins (TPs) of Pinot Noir and Marselan seed extracts are high. These parameters for the Pinot Noir extract were as follows: TPC—111.22 mg GAE/g DW, TFs—51.50 mg QE/g DW, and TPs—170.45 mg CE/g DW. Similar results were obtained for the Marselan extract: TPC—103.24 GAE/g DW, TFs—52.01 mg QE/g DW, and TPs—152.18 mg CE/g DW. TPC, TFs, and TPs values were higher in the Pinot Noir and Marselan seed extracts compared to the other two types of grape seed extracts (Figure 2). All these compounds provide high antioxidant capacity. The DPPH method determined that the antioxidant capacity of Pinot Noir extract was 579.33 µM TE/g DW. That of Marselan extract was 597.23 µM TE/g DW. When comparing these results with the data obtained by other authors, it is obvious that both extracts are rich in polyphenols [4,10]. Rockenbach et al. [43] indicated that the TPC of Pinot Noir seed extracts was 165.18 mg CE/g DW. Guaita and Bosso [44,45,46] described that the values of the TPC of seed extracts of four red grape cultivars varied from 73.7 to 107.8 mg GAE/g DW, and TF values for GSEs were in the interval 6.90–25.91 mg QE/g DW. The TPs C values of the studied extracts are comparable to those obtained by other authors for seed extracts of different red grapes (85.2–152.0 mg CE/g DW) [5,47]. The data of individual compounds of Pinot Noir and Marselan seed extract showed that the two extracts have high concentrations of catechin, epicatechin, and different types of procyanidins (B1, B2, B3, and C) [39]. The Pinot Noir and Marselan seed extracts were chosen for further investigation because of their high polyphenol content.

Evaluating the nutritional value and macro- and micronutrient content of Pinot Noir and Marselan grape seed flours and extracts is essential for their application. The ash, moisture, protein, fat, fiber, and carbohydrate content in them was investigated. It was found that the chemical compositions of the flours and seed extracts of the two grape varieties are different (Table 2). The ash content in the flour from the studied grape seeds is about 1.6 times higher than that in the extracts. The reason for the lower results in the extracts is probably that the extraction was carried out with 70% ethyl alcohol, and the extraction of minerals is weaker. Similar percentages of ash in flour and extract have been obtained by other authors [4,17,21,22]. Beres et al. [45,48] carried out the extraction with warm distilled water and obtained a higher percentage of ash in their extract. The protein content in the seed flours is almost twice as high as in the extracts. The probable reason is that a large part of the proteins remains in the ultrastructure of the cell wall. The situation is similar with dietary fibers—their content in the extracts is lower than in the flours. It is important to say that the fibers in the extracts are soluble dietary fibers, which is a desirable characteristic for biologically active substances. The opposite result is observed for carbohydrates. Their content is about 2–2.5 times higher in both extracts compared to in the flours. The lipid content in both flours (15–16%) is also slightly higher than in the extracts (9–11%). Similar results have been obtained by other authors [17]. They found that the percentage of fats in red grape seed extracts is of the same order. In Table 2, it is obvious that the results of all studied indicators of the flour and extract in both types of grape seeds are very close.

The minerals composition of grape seed flours and extracts is summarized in Table 3. In general, the results of all the studied indicators in Pinot Noir grape seed flour and extract are slightly higher compared to those in the samples of Marselan grape seeds. Based on these studies, it was found that the flour and extract of the seeds of both types of grapes are rich in the macroelements potassium, phosphorus, magnesium, calcium, and sodium as well as in microelements. The microelement amounts of zinc, copper, and iron in grape flours was higher than in grape seed extracts. Similar results for the microelements in grape flours and extracts have been published by other authors [20,21]. Their content is very high compared to those contained in other plant extracts [42]. The obtained results show that grape seed flours and extracts can be successfully used as natural supplements and food additives. Typically, fiber-rich grape seed flours are more suitable for use as additives to produce functional foods than extracts. The only drawback is their insolubility, but in some food products, such as bread, confectionery, etc., they can be directly added [18,21]. The extracts also have good nutritional value and a good mineral profile but have the advantage of containing soluble dietary fiber. In addition, thanks to their extraction with 70% ethyl alcohol, the extracts are rich in various phenolic compounds. The latter provide the antioxidant, antilipid, and antimicrobial effects of the extracts. For this reason, grape seed extracts are widely used as supplements and additives for functional foods [22,23,24]. Furthermore, the phenolic compounds contained in the supplements possess inhibitory properties toward certain enzymes such as α-amylase, α-glucosidase, pancreatic lipase, cholinesterase, cyclooxygenases, and 5-lipohygenase, making them even more valuable products capable of being used to manage the following diseases: diabetes, obesity, Alzheimer’s disease, and skin hyperpigmentation [9,26].

The evaluation of the biological properties of dietary supplements is another important feature. Diabetes and obesity are significant problems facing modern society. The enzymes salivary α-amylase and α-glucosidase are responsible for the digestion of dietary carbohydrates. The inhibitory effect of Pinot Noir and Marselan grape seed extracts on salivary α-amylase, α-glucosidase, and pancreatic lipase was studied (Figure 3, Figure 4 and Figure 5 and Table 4). The results show that both in buffer and in salt solution, α-glucosidase is much more sensitive to GSE’s inhibition activity than the other two enzymes. Moreover, in the case of α-glucosidase, the reaction occurs only in the presence of all three components—enzyme, substrate, and extract—while α-amylase and pancreatic lipase require pre-incubation with the extract and the subsequent addition of the substrate. It is likely that the inhibition of α-glucosidase occurs by a different mechanism. The extract probably inhibits the enzyme–substrate complex, but this assumption requires further research. When comparing the results obtained in buffer and in salt solution, a weaker degree of inhibition of all three enzymes in salt solution is noted, as are correspondingly higher IC_50_ values. Obviously, salts do not have a positive effect on enzymes’ degree of inhibition. The IC_50_ results were compared to those of commercial synthetic drugs used for diabetes (Acarbose) and obesity (Orlistat). Acarbose is an alpha-glucosidase inhibitor used for the management of glycemic control in patients with type 2 diabetes. Orlistat is used to manage obesity by reversibly inhibiting gastric and pancreatic lipases within the gut. In Table 4, it is obvious that the inhibitory potential of these two drugs is weaker compared to the two extracts. Results regarding the inhibitory potential of the two studied extracts were compared with results obtained by other authors. It was found that the degree of inhibition of the three enzymes in some cases was similar [31], but in other cases, inhibition was higher [33,39].

From the obtained results, it is obvious that the degree of enzyme inhibition is greater in the Marselan extract than in Pinot Noir. Naturally, the degree of inhibition of these enzymes depends greatly on the type and content of polyphenols in these extracts. In our previous paper [39], the individual composition of the phenolic compounds in both extracts was determined by the RT-HPLC method. The following compounds in these extracts were detected: gallic acid; catechin; epicatechin; epigallocatechin gallate; procyanidin B_1_, B_2_, and B_3_; procyanidin C_1_; oenin; and quercetin. The amount of catechin, epicatechin, and procyanidins was very high in the extracts. It is noteworthy that the amount of epicatechin in the Marselan extract (14.27 mg/g) was higher compared to the result for the Pinot Noir extract (10.16 mg/g) [39]. The flavonoid values for the Marselan extract were also slightly higher than those for the Pinot Noir extract. The amounts of individual procyanidins in both extracts were similar. It is likely that the greater amounts of total flavonoids and epicatechin lead to Marselan seed extract having a higher inhibitory potential than Pinot Noir seed extract. In addition, the use of ethanol for extraction is much more suitable than hot water because the extract is significantly enriched with phenolic compounds and its inhibitory potential increases. Hasan [31] has proven that the inhibitory potential of the ethanol extract is about three times stronger than the aqueous extract of the same type of grape seeds.

A number of studies have been published demonstrating the influence of phenolic composition on GSE’s inhibitory potential against food enzymes [27,46]. Its digestive enzyme inhibitory activity depends on the molecular weight, number, and position of substitution of phenolic compounds [47]. Flavonoids are well known as α-amylase inhibitors [48,49]. Tadera et al. [50] found that α-amylase inhibition potency is correlated with the number of hydroxyl groups of flavonoids. Other authors have described that procyanidins are very important for enzymes’ inhibition. They have more potential interaction sites than monomeric phenolic compounds and can easily crosslink enzymes [51,52]. Buchholz et al. [53] studied the lipase inhibitory activity of 70 different flavonoids. They suggested that the inhibitory effect depends on the number and position of phenolic hydroxyl groups. All these studies prove that the high content of flavonoids and procyanidins in Pinot Noir and Marselan extracts determines their high inhibitory potential against α-amylase, α-glucosidase, and pancreatic lipase. The better inhibitory potential of Marselan can be explained by its higher content of epicatechin and total flavonoids and its high antioxidant potency compared to Pinot Noir extract.

In vitro experiments were conducted to study the impact of digestive conditions on GSE’s inhibitory potential against the three enzymes. Table 5 shows that the inhibition percentage of α-glucosidase with undigested Pinot Noir extract dissolved in salt solution was 1.5 times lower compared to the digested extract. In the case of Marselan extract, this difference was smaller, but again, in the digested extract, the percentage of enzyme inhibition was higher. The higher results with digested Marselan extract compared to digested Pinot Noir extract are analogous to the results in buffer and salt solution. This may be due to the higher content of flavonoids in the Marselan extract. This trend is repeated for pancreatic lipase. These results clearly show that the chemical transformation induced by the stomach and intestines has a significant influence and positive effect on the inhibitory effect of the extracts. In addition, it is obvious that the in vitro evaluation of the extracts’ inhibitory properties on α-glucosidase and pancreatic lipase may underestimate the real in vivo potential of the extracts. In fact, the obtained data suggest that the extracts may partially act as a prodrug whose activity gradually increases during intestinal passage. Similar results have been obtained by other authors [47,53,54,55]. Therefore, the obtained data suggest that the consumption of grape extracts may help regulate postprandial glucose levels in patients with T2D and fat levels in obese patients.

Grape seeds are a by-product of wine production, and they are a cheap source for obtaining valuable bioactive compounds. According to the findings of the current research, together with our previous papers, a full assessment of Pinot Noir and Marselan GSEs has been made from the perspective of physicochemical characteristics, polyphenolic contents, antioxidant potential, nutritional value, macro-and microelements content, and functional value. The novelty of this study is the evaluation of the inhibition potential of Pinot Noir and Marselan GSEs against three key digestive enzymes and their proximate composition. The obtained results show GSE’s high potential for application as dietary supplements for the management of diabetes and obesity. However, for a definitive assessment of this potential, it is necessary to conduct in vivo research.

## 4. Materials and Methods

### 4.1. Materials and Chemicals

The materials used in this study were the wastes (pomaces) from the vinification of two red wines from *Vitis vinifera* L. cv. Pinot Noir and Marselan grapes (Pink Pelikan Winery Ltd., Silistra, Bulgaria). These grape types are grown in the Danube region, near the city of Ruse, Bulgaria. Pink Pelikan Winery Ltd., according to a list of the Ministry of Agriculture and Food, Executive Agency for Vine and Wine, has the unique identification number 3688. It is an old winery, and their grape types were identified. Their varieties have a protected designation of origin (PDO). The Pinot Noir variety is originally from Burgundy. In the Vitis International Variety Catalogue (VIVC), Pinot Noir has the variety number 9279 and national variety ID BGR100. The Marselan variety was obtained by the INRA in 1961 and was the result of crossbreeding Cabernet Sauvignon and Grenache. The International Marselan variety number is 16,383, and the national variety ID is BGR062. Both grape varieties are recommended for the geographical Danube region. Variety identification was conducted by the Institute of Viticulture and Enology, Agricultural Academy, Ministry of Agriculture and Food. The identification was conducted by traditional and molecular ampelography. According to the official variety list of varieties of wines from the Executive Agency for Variety Testing, Field Inspection and Seed Control, Ministry of Agriculture, the Pinot Noir and Marselan were recognized for certification and trade in the territory of Republic of Bulgaria in 2023. The Association of Manufacturers and Nurserymen of Table Grapes is the variety maintainer organization for these grape varieties in Bulgaria.

Hydrochloric acid (37% *w*/*w*), ethanol, sodium hydroxide, p-nitrophenyl-α-D-glucopyranoside (p-NPG), 3,5-dinitrosalicylic acid (DNS), sodium potassium tartrate, bile salts, sodium sulphate, sodium hydrogen sulphate, sodium chloride, sodium carbonate, potassium chloride, potassium thiocyanate, hydrogen peroxide, urea, sodium bicarbonate, ammonium carbonate, olive oil, and gum arabic were purchased from Sigma–Aldrich Chemie, GmbH, Steinheim am Abbuch, Germany. The α-amylase from human saliva (173 U/mg), α-Glucosidase from Saccharomyces cerevisiae, Type I, 20.7 U/mg), lipase from porcine pancreas (15,900 U/mg), pancreatin from porcine pancreas (250 U/mg), pepsin from porcine gastric mucosa (lyophilized powder, 2500 U/mg), acarbose, and orlistat were purchased from Sigma–Aldrich Chemie GmbH, Taufkirchen, Germany. All aqueous solutions were prepared with pure water generated by a Milli-Q water (18.2 MW) device (Merck, Darmstadt, Germany).

### 4.2. Preparation of Grape Seed Extract

The seeds were separated from the skin using a plastic sieve. Then, the treatment of the grape seeds was carried out separately, with processing by washing and drying at 40 °C for 14 h and storage at 4 °C. For each experiment, a certain quantity of dry grape seeds was ground to a powder with a diameter of 2.5–22.5 µm. A mixture of grape seed powder (5 g) with 25 mL of 70% aqueous ethanol was stirred using an MMS-3000 magnetic stirrer (Boeco, Hamburg, Germany) at a constant rate of 500 rpm at ambient temperature and pressure for 3 h. The upper liquid was separated, and the process was repeated with another 25 mL of 70% aqueous ethanol. The resulting mixture was centrifuged at 6000× *g* rpm for 10 min. The supernatant was separated and concentrated to 1 mL in a vacuum evaporator (Rotavapor R-215, Buchi, Flawil, Switzerland) in a water bath at 50–60 °C and 100–175 hPa vacuum pressure.

### 4.3. Chemical Analysis of Grape Seed Extracts and Flours

The Proximate Composition Analysis of the two grape varieties’ seed flour and grape seed extract from the ash, crude protein, crude fat, total carbohydrate, and total dietary fiber contents was carried out using AOAC methods. The moisture of the samples was determined by drying them at 100 °C to a constant weight AOAC Method 934.01 [56]. The crude fat content was determined by a modified AOAC Method 945.16 [56] with petroleum ether as a solvent using FOSS Soxtec TM 2050 Extractor (FOSS, Hoganas, Sweden). The ash content was determined using the AOAC Method 923.03 [56] with a Barnstead Thermolyne 30,400 muffle furnace (Barnstead International, Dubuque, IA, USA). The protein content was determined by modified Kjeldahl method that determines total nitrogen in nitrate-containing materials and animal feeds, AOAC 976.05 [56]. The sample is digested in sulfuric acid; ammonia is distilled; and excess acid is titrated. The nitrogen-to-protein conversion factors used for both samples was 5.6 [57].

Total dietary fiber was determined by the enzymatic gravimetric methodology AOAC 993.19 [56]. Total dietary fiber was determined by gelatinizing duplicate samples (previously fat-extracted if the fat content was greater than 10%) with heat-stable alpha-amylase, digesting with protease and amyloglucosidase to remove protein and starch, and diluting the aqueous digest with four volumes of ethanol to precipitate soluble dietary fiber. The residue was filtered; washed with 78% ethanol, 95% ethanol, and acetone; dried; and weighed. All the analyses were replicated three times. Independently replicated digestions were performed for each experiment.

Total carbohydrates were determined by difference using the following equation:

% carbohydrates = 100 − (% moisture + % protein + % ash + % fat + % fiber)

### 4.4. Determination of Mineral Composition

The samples were ground and sieved through pores with φ < 500 µm. Each sample was weighed (0.25 g) with a precision up to 0.0001 g and quantitatively transferred to a Teflon container, which was left with an open lid for 24 h. The next day, 2 mL of hydrogen peroxide was added, and the Teflon container lid was tightly closed. Two blank samples were prepared in a similar manner. The blank samples were decomposed simultaneously with the test samples in the PreeKem microwave system, model M6 with a rotor H16. The microwave system programming included the following five steps: (1) temperature 100 °C, 300 W, with 5 min to reach the temperature and 5 min to maintain it; (2) temperature 100 °C, 350 W, with 8 min to reach the temperature and 3 min to maintain it; (3) temperature 120 °C, 650 W, maintained for 5 min; (4) temperature 120 °C, 900 W, maintained for 3 min; and (5) temperature 180 °C, 1350 W, maintained for 3 min. After the completion of the decomposition, the samples were left for 10 min to cool the system. Then, the vessels were opened, and the caps were carefully washed with ultrapure water. The resulting extract was filtered through a blue-ribbon filter and quantitatively transferred into a 100 mL volumetric flask. The extraction solutions were then measured on a Thermo Scientific iCAP Q ICP-MS apparatus, Bremen, GmbH, Germany.

### 4.5. α-Amylase Inhibitory Activity of GSE

The α-amylase inhibitory activity of GSE was determined according to the method described by Lu et al. [58], with modifications. An amount of 0.2 mL of α-amylase (15 U/mL in 0.1 M PB 6.9) solution was briefly pre-mixed with 0.2 mL of GSE at different concentrations (from 0.001 to 0.3 mg/mL) and incubated at 37 °C for 20 min. Then, 0.2 mL of starch solution (0.5%) was added as the substrate and incubated for 5 min. Next, 1 mL of DNS reagents (10 mg/mL 3, 5-dinitrosalicylic acid and 120 mg/mL sodium potassium tartrate in 0.4 mol/L sodium hydroxide) were added to terminate the reaction and heated for 10 min at 100 °C. After cooling down, the absorbance was measured at 540 nm. The α-amylase inhibitory activity was calculated as follows:

Inhibition (%) = [(A control − A sample)/A control] × 100

To prepare the control sample, instead of GSE, 0.2 mL of buffer was added. Acarbose was used as a comparative sample. Independent replicates were performed three times.

### 4.6. α-Glucosidase Inhibitory Activity of GSE

The α-glucosidase inhibitory activity was determined according to the method of Xu et al. [59] with slight modifications. A total of 600 μL of phosphate buffer (0.1 M, pH 6.8), 100 μL of GSE (from 0.0025 to 0,3 mg/mL in phosphate buffer, 0.1 M, pH 6.8), 100 μL α-glucosidase (0.6 U/mL), and 200 μL pNPG (5 mM) as substrate were briefly mixed and incubated for 20 min at 37 °C. Then, 1 mL of sodium carbonate (0.1 M) was added to each sample, and absorbance was measured at 410 nm. The α-glucosidase inhibitory activity was calculated as follows:

Inhibition (%) = [(A control − A sample)/A control] × 100

To prepare the control sample, instead of GSE, 0.1 mL of buffer was added. Acarbose was used as a comparative sample. The analysis was replicated three times.

### 4.7. Pancreatic Lipase Inhibitory Activity of GSE

The pancreatic lipase inhibitory activity was determined according to the method of Jagdish et al. [60]. A total of 200 µL of pancreatic lipase with a concentration of 1 mg/mL was added to 200 µL of GSE with different concentrations (from 0.05 to 2 mg/mL). The mixture was incubated at 37 °C for 20 min, with stirring. After that, a 5 mL emulsion of 5% olive oil (in aqueous 3% gum arabic) was heated at 60 °C and stirred with a magnetic stirrer for 15 min. The enzyme–extract mixture was added to the emulsion, and the mixture was incubated for 30 min at 37 °C, with stirring. Then, the latest mixture was added to 10 mL of ethanol with 2 drops of 1% phenolphthalein. The mixture was titrated with 0.05 M NaOH. Orlistat was used as a comparative sample. The analysis was replicated three times.

### 4.8. In Vitro Digestion Method

The simulated gastrointestinal digestion of GSE in vitro was performed according to the procedure reported by Tenore et al. [35], with slight modifications. In brief, to simulate the oral phase, 5 mL of artificial saliva medium (89.6 g/L KCl, 20.0 g/L KSCN, 88.8 g/L NaH_2_PO_4_, 57.0 g/L Na_2_SO_4_, 175.3 g/L NaCl, 84.7 g/L NaHCO_3_, and 2.0 g/L urea) was diluted with 45 mL of distilled water; the pH was adjusted to 6,8, with 0.1 mol/L HCI; and salivary α-amylase (4.3 mg) was added. After that, GSE was added (4 mg/mL), and the mixture was homogenized in a thermostat shaker (Honour Instrument Co., Ltd., Tianjin, China) at 37 °C for 5 min. Then, 10 mL of saliva medium were transferred to a new test tube and kept in boiling water for 5 min to stop the reaction. After cooling down, the pH of the medium in the test tube was adjusted to neutral, the sample was suitably diluted and was used for the analysis of α-amylase by the method described in 3.5. For the gastric digestion, the pH of the remaining incubation medium was immediately adjusted to 2.0. HCl (6 mol/L) and pepsin (12 mg) dissolved in 1 mL HCl (0.1 mol/L) were added to start the gastric digestion. The mixtures were then incubated at 37 °C for 2 h. After the gastric digestion, 10 mL of gastric medium was removed, and the pH was adjusted to 7.0 with NaOH (0.1 N) to stop the enzymatic reaction. After suitable dilution, the fraction was tested for inhibitory activity against α-glucosidase and pancreatic lipase by the method described in 3.6 and 3.7. For the intestinal digestion, the pH of the remaining gastric medium was adjusted to 6.5 with NaHCO_3_ (0.5 mol/L), and a 5 mL mixture of pancreatin (8 mg/mL) and bile salts (50 mg/mL) was added to the solution and incubated for another 2 h at 37 °C. Then, 10 mL of intestinal medium was removed, and after suitable dilution, the fraction was tested for inhibitory activity against α-glucosidase and pancreatic lipase. Three independently replicated digestions were performed.

### 4.9. Statistical Evaluation

The obtained results were statistically evaluated by the two-way ANOVA method for the analysis of variance using the SAS computer program, version 9.1.3, Cary, NC, USA. The results were presented as mean ± standard deviation. Each test was performed three times (*n* = 3).

## 5. Conclusions

The high nutritional values and levels of macro- and microelements that were obtained convincingly show the high potential of Pinot Noir and Marselan grape seed flours and extracts for application as food additives and supplements. The high polyphenol content provides a high DPPH antioxidant capacity, 579.33 µM TE/g DW in Pinot Noir extract and 597.23 µM TE/g DW in Marselan extract. In buffer, the grape extract rich in flavonoids and procyanidins presented high inhibition potential against salivary α-amylase (IC_50_ 11.18 ± 2.92 µg/mL), α-glucosidase (IC_50_ 2.53 ± 0.24 µg/mL), and pancreatic lipase (IC_50_ 601.28 ± 65.58 µg/mL). In vitro digestion experiments increased the GSE’s inhibitory potential against the studied enzymes 1.5-fold compared to the undigested extract. The results prove GSE’s high potential for application as dietary supplements, which can lead to the management of diabetes and obesity.

## Figures and Tables

**Table 1:** Color evaluation of seed flour and extract of Pinot Noir and Marselan grapes.

Grapes	GSF and GSE	Lightness (L* Value)	Redness (a* Value)	Yellowness (b* Value)
Pinot Noir	flour	22.45 ± 1.56	2.29 ± 0.21	4.95 ± 0.42
	extract	41.84 ± 1.82	4.21 ± 0.39	21.78 ± 1.43
Marselan	flour	28.25 ± 2.10	3.63 ± 0.35	1.81 ± 0.15
	extract	43.36 ± 2.42	14.10 ± 0.98	19.88 ± 1.37

Values represent the average ± standard deviation of triplicate experiments (*n* = 3). GSE—grape seed extract. GSF—grape seed flour.

**Table 2:** Nutrient composition of Pinot Noir and Marselan grape seed flours and extracts.

Parameters, %	Pinot Noir		Marselan	
	GSE	GSF	GSE	GSF
Ash	2.32 ± 0.11	3.42 ± 0.16	2.15 ± 0.06	3.61 ± 0.15
Moisture	7.34 ± 0.45	6.45 ± 0.49	6.98 ± 0.25	6.68 ± 0.43
Crude protein	8.98 ± 0.55	15.26 ± 0.65	7.12 ± 0.41	16.87 ± 0.67
Crude fat	9.12 ± 0.61	15.82 ± 0.55	11.32 ± 0.67	16.41 ± 0.66
Total dietary fiber	29.25 ± 1.23	32.05 ± 1.72	28.42 ± 1.02	34.02 ± 1.75
Total carbohydrates	59.18 ± 4.11	27.09 ± 1.24	60.83 ± 4.23	22.45 ± 0.95

Values represent the average ± standard deviation of triplicate experiments (*n* = 3). GSE—grape seed extract. GSF—grape seed flour.

**Table 3:** Mineral profiles of Pinot Noir and Marselan grape seed flours and extracts.

Elements	LOD, mg/kg	Pinot Noir GSF	Pinot Noir GSE	Marselan GSF	Marselan GSE
		mg/kg DW	mg/kg DW	mg/kg DW	mg/kg DW
Na	34.70	194.98 ± 3.25	542.04 ± 22.32	120.77 ± 0.30	168.47 ± 5.84
Mg	30.79	1401.17 ± 56.68	461.87 ± 15.95	1464.12 ± 92.96	264.65 ± 15.18
Al	5.48	112.02 ± 1.80	44.01 ± 0.99	153.22 ± 4.20	16.43 ± 1.23
P	8.10	3049.94 ± 25.95	3693.10 ± 25.08	3821.53 ± 263.95	1636.96 ± 10.01
K	18.84	5108.01 ± 122.64	5279.99 ± 113.16	3718.25 ± 214.39	4030.24 ± 4.89
Ca	93.73	1853.80 ± 74.50	257.53 ± 49.91	1878.77 ± 87.96	188.59 ± 3.88
Cr	0.20	0.68 ± 0.02	4.44 ± 0.17	0.88 ± 0.08	0.87 ± 0.02
Fe	4.37	160.89 ± 2.91	119.07 ± 2.08	78.59 ± 1.41	31.60 ± 1.79
Zn	1.98	19.75 ± 0.56	14.72 ± 0.25	12.09 ± 0.55	3.19 ± 0.22
Cu	0.30	19.65 ± 2.18	17.84 ± 0.90	10.13 ± 0.13	9.32 ± 0.05

Values represent the average ± standard deviation of triplicate experiments (*n* = 3). GSE—grape seed extract. GSF—grape seed flour. DW—dry weight.

**Table 4:** IC_50_ value (µg/mL) of GSE in buffer and salt solution against α-amylase, α-glucosidase, and pancreatic lipase.

Enzymes	Pinot Noir GSE		Marselan GSE		Commercial Inhibitor
	Buffer	Salt Solution	Buffer	Salt Solution	
α-amylase	99.05 ± 0.82	451.74 ± 42.52	11.18 ± 2.92	12.17 ± 1.32	Acarbose 2381 ± 211.23
α-glucosidase	8.53 ± 0.74	14.16 ± 1.25	2.53 ± 0.24	2.56 ± 0.27	
Pancreatic lipase	601.28 ± 62.15	725.76 ± 71.85	601.28 ± 65.58	725.53 ± 69.17	Orlistat 1150 ± 91.35

Values represent the average ± standard deviation of triplicate experiments (*n* = 3). GSE—grape seed extract.

**Table 5:** α-glucosidase and pancreatic lipase inhibition activity (%) of digested GSE.

Enzymes	GSE Concen- tration, mg/mL	Pinot Noir GSE			GSE Concen- tration, mg/mL	Marselan GSE		
		Gastric Phase	Intestinal Phase	Salt Solution		Gastric Phase	Intestinal Phase	Salt Solution
α-glucosidase	0.001	49.23 ± 2.95	42.38 ± 2.04	1.71 ± 0.11	0.0001	38.52 ± 2.15 4	36.27 ± 1.27 2	32.12 ± 1.11
	0.0025	72.47 ± 5.23	70.26 ± 4.92	50.33 ± 3.12	0.001	72.46 ± 5.14	70.35 ± 4.92	69.48 ± 4.82
	0.030	88.54 ± 6.54	90.56 ± 6.68	82.32 ± 6.18	0.005	95.67 ± 6.73	94.22 ± 6.65	91.26 ± 6.75
Pancreatic Lipase	0.50	22.28 ± 1.12	20.18 ± 0.90	17.24 ± 1.17	0.50	35.56 ± 1.28	30.42 ± 1.27	25.24 ± 1.14
	0.80	45.36 ± 2.13	40.48 ± 1.92	32.38 ± 1.14	0.80	52.31 ± 2.36	50.11 ± 2.93	42.48 ± 1.92
	1.50	82.69 ± 5.92	80.67 ± 6.10	70.56 ± 5.03	1.50	85.25 ± 6.33	80.54 ± 5.96	74.36 ± 5.34

Values represent the average ± standard deviation of triplicate experiments (*n* = 3). GSE—grape seed extract.

## Data Availability

Data are contained within the article.

## Abbreviations

The following abbreviations are used in this manuscript:

GSE	Grape seed extract
GSF	Grape seed flour
FDA	Food and Drug Administration
DPPH	2,2 diphenyl-1-picrylhydrazyl
TFs	Total flavonoids
TPC	Total phenolic content
TPs	Total procyanidins
DW	Dry weight
